# Implementation of a novel color additive for bleach disinfectant wipes enhances cleaning performance in an academic medical center

**DOI:** 10.1017/ice.2023.61

**Published:** 2023-12

**Authors:** Kevin Tyan, Jason Kang

**Affiliations:** Kinnos Inc, Brooklyn, New York

## Abstract

Effective hospital environmental cleaning requires proper technique and training. Highlight is a novel additive that colorizes bleach wipes to help visualize wiped surfaces and fades to colorless to confirm effective cleaning. In a 401-bed hospital study, Highlight reduced fluorescent marker removal failure rates from a baseline of 12.4% to 0.6%.

Contaminated environmental surfaces in healthcare facilities are an important source of infectious disease transmission.^1^ These healthcare-associated infections (HAIs) result in up to $45 billion in direct costs and affect nearly 2 million patients in the United States each year.^2^ One key strategy for mitigating the spread of HAIs is environmental cleaning of high-touch surfaces using an effective disinfectant, thorough cleaning technique, and proper training. When implemented properly, enhanced cleaning interventions can be a powerful tool to control outbreaks and reduce costs. For example, the introduction of an environmental cleaning bundle across 11 Australian hospitals, including improved cleaning technique and product use, resulted in decreased vancomycin-resistant enterococci (VRE) and *Staphylococcus aureus* infections and a net monetary benefit of USD $680,000.^3^ Similarly, a simulation of a 200-bed model hospital during a 1-year period found that enhanced daily cleaning was the most cost-effective infection control intervention against *C. difficile* transmission, saving $358,268 and 36.8 quality-adjusted life-years annually.^4^

However, there are significant challenges in ensuring the effectiveness of environmental cleaning. The labor-intensive and often repetitive nature of manual cleaning raises the possibility of human error. In particular, disinfectant wipes are susceptible to a range of improper usage, including incomplete disinfectant coverage on the surface, cross contamination from wiping multiple surfaces with the same wipe, and noncompliance with stated contact times.^5^ Most importantly, certain high-touch surfaces are inconsistently cleaned compared to other objects. In a study across 23 hospitals, overall thoroughness of cleaning was only 49%; bedpan cleaners, door knobs, and light switches were cleaned <25% of the time.^6^ Thus, there is an important need for new environmental cleaning strategies to enable environmental services (EVS) workers to achieve effective cleaning performance with proper technique.

One potential method to enhance cleaning technique and improve thoroughness of disinfection is through a real-time visual feedback mechanism. Highlight (Kinnos, Brooklyn, NY) is a blue dye indicator that colorizes bleach disinfectant wipes, enabling EVS staff to visually confirm which surfaces have been wiped and assess how thoroughly the disinfectant is spread across the surface. This bright blue trace then fades away to colorless in minutes to prevent surface residue and eliminate the need for further cleanup. The color additive is dispensed onto bleach wipes through a lid device that attaches onto commercially available bleach-wipe containers, as previously described.^7,8^ Thus, the color additive confers an advantage to traditional disinfectants, which are transparent in color and difficult to visualize when applied to a surface. Other potential benefits to this indicator system include immediate visual feedback on wiping technique by revealing which parts of a surface are incompletely wiped, as well as preventing interference with a surface before wet-contact time is achieved.

## Methods

To evaluate whether the implementation of a color additive with bleach disinfectant wipes improves cleaning performance, we conducted a prospective study at a 401-bed academic medical center (Keck Hospital of USC, Los Angeles, CA) from October 2020 to June 2022. During this period, terminal or daily cleaning performance was evaluated in a random sample of patient rooms across 3 medical inpatient wards. Phases of this study were delineated by every 2 calendar quarters for the baseline collection and every 3 calendar quarters for the implementation phase to enable comparisons across similar time frames. During the baseline phases, from Q4 2020 to Q1 2021 (baseline phase 1) and from Q2 2021 to Q3 2021 (baseline phase 2), EVS staff performed cleaning of patient rooms using standard bleach disinfectant wipes (Clorox Healthcare Bleach Germicidal Wipes, Clorox, Oakland, CA). During the implementation phase from Q4 2021 to Q2 2022, cleaning was performed using the color additive in combination with standard bleach wipes. During all phases, EVS staff followed standard procedures and instructions as stated on standard bleach wipes, including following a precleaning step to remove heavy soil loads as indicated. In total, 36 EVS staff were involved in the cleaning performed during this study, including 11 staff members who were evaluated across multiple quarters. Immediately prior to EVS cleaning of a patient room, an infection control staff member randomly applied fluorescent marking gel (DAZO; Ecolab, Saint Paul, MN) on as many as 23 high-touch surfaces. After unsupervised cleaning of the patient room by an EVS worker, the infection control staff member performed a black light assessment and assigned a “pass” or “fail” score for each high-touch surface based on complete removal of fluorescent marker.

## Results

In total, 3,288 high-touch surfaces were sampled during this study (1,014 in baseline phase 1, 714 in baseline phase 2, 1,560 in the implementation phase), with an average of 143 data points collected from each of the 23 designated high-touch surfaces. The most frequently sampled object was the room light switch (156 samples, 4.7%), whereas the least sampled surface was underneath the bedside table (84 samples, 2.6%). Of the 69 patient rooms that were assessed during this study, cleaning assessment was performed a median of 2.3 times (range, 1–8).

**Fig. 1.**
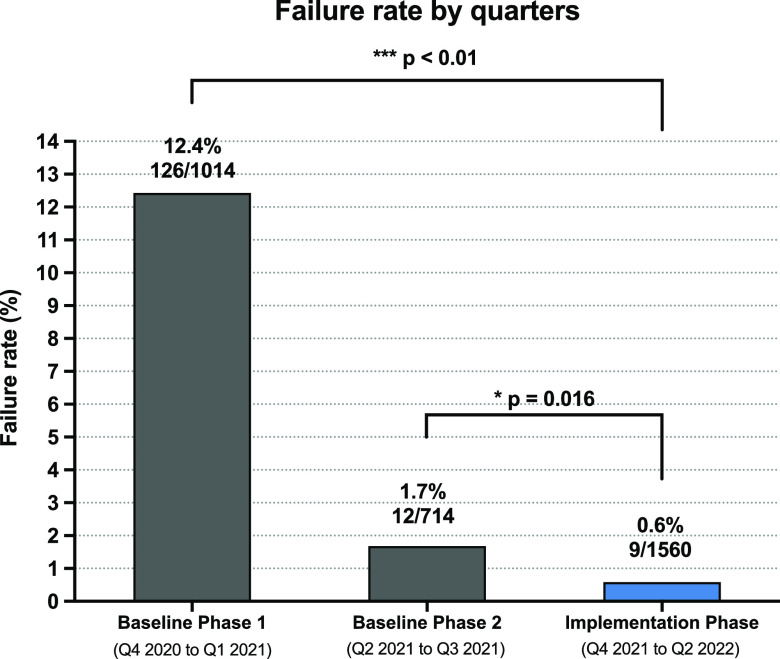
Comparison of cleaning failure rate between baseline and color additive phases. Disinfection quality was assessed by fluorescent marker removal. The baseline phase 1 was from Q4 2020 to Q1 2021 and the baseline phase 2 was from Q2 2021 to Q3 2021. The color additive was implemented from Q4 2021 to Q2 2022 (implementation phase). Numbers above each bar indicate the failure rate based on number of unremoved fluorescent marker samples over the total number of fluorescent marker samples during that phase.

During the baseline phases, cleaning with bleach wipes alone resulted in higher failure rates compared to cleaning with the color additive. In baseline phase 1, the failure rate was 12.4% (126 of 1,014 samples) and in baseline phase 2 the failure rate was 1.7% (12 of 714 samples). In comparison, cleaning with the color additive during the implementation phase reduced the failure rate to 0.6% (9 of 1,560 samples). Figure 1 illustrates this comparison between phases, with a statistically significant reduction in failure rates during the trial phase compared to baseline phases.

**Fig. 2.**
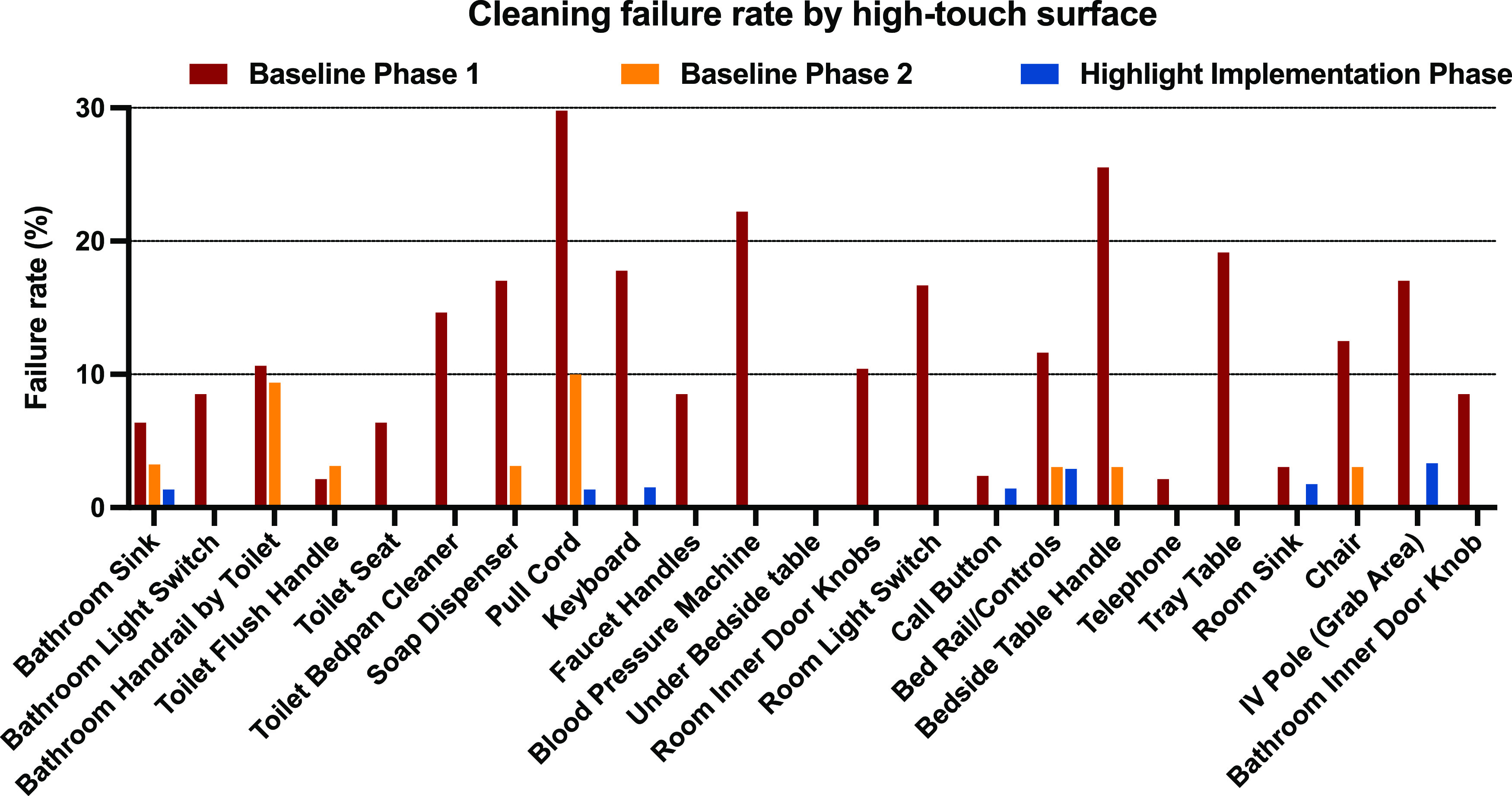
Comparison of cleaning failure rate between high-touch surfaces. Rates of cleaning for each of the 23 high-touch surfaces as measured by fluorescent marker removal. Failure rates are shown for the baseline phases (baseline phase 1, Q4 2020 to Q1 2021, shown in red; baseline phase 2, Q2 2021 to Q3 2021, shown in orange) and when the color additive was implemented (implementation phase, Q4 2021 to Q2 2022, blue). Failure rates are based on number of failed samples over total samples.

In addition, we detected significant variability in thoroughness of cleaning across different types of high-touch surfaces (Fig. 2). For example, high failure rates in baseline phase 1 were driven by poor cleaning of the pull cord (29.8%), blood-pressure machine (22.2%), and bedside table handle (25.5%), whereas other objects, including the toilet flush handle (2.1%) and telephone (2.1%), were more consistently cleaned. Over the next 2 phases of the study, improvements in cleaning were observed across all high-touch surfaces, especially during the implementation phase in which there were no failures recorded for 16 of 23 high-touch surfaces.

## Discussion

These results demonstrate that the implementation of a color additive to enhance visualization of bleach disinfectant wipes can improve thoroughness of cleaning. These data are consistent with previous hospital studies of the color additive, including in a 500-bed academic center in which ATP failure rate was reduced from 5.7% to 0%,^7^ as well as in a 781-bed academic center in which failure rates were reduced from 15.0% to 4.5% (fluorescent marker removal) and from 3.6% to 2.5% (ATP assay).^9^ This study of the color additive features the largest sample size of high-touch surfaces thus far (N = 3,288), with detailed insights into cleaning performance across 23 types of surfaces. In addition, the large number of EVS workers working across different inpatient units has improved the generalizability of our results. Overall, we have demonstrated that ineffective cleaning and disinfection was partly driven by inconsistent wiping of certain types of high-touch surfaces and that the implementation of the color additive significantly improved cleaning performance across all types of surfaces. Compared to the baseline periods (baseline phase 1 and baseline phase 2), EVS workers using the color additive achieved 95% and 66% reductions in fluorescent marker failure rates during the implementation phase.

This study had several limitations. Because cleaning performance was evaluated on a convenience sample of rooms and staff that completed daily or terminal cleaning, we did not randomize EVS staff between different phases of the study. As a result, we did not control for whether specific workers may have contributed to better or worse cleaning performance. Thus, it is important to consider the possibility of continuous worker improvement over time as a contributor to decreased failure rates. Future studies of this colorized cleaning approach should also assess other factors, including its impact on rates of HAIs, usability feedback, and patient satisfaction.
